# Mass Spectrometry-Based Proteomics for Assessing Epitranscriptomic Regulations

**DOI:** 10.1002/mas.21911

**Published:** 2024-10-18

**Authors:** Yen-Yu Yang, Zhongwen Cao, Yinsheng Wang

**Affiliations:** 1 Department of Chemistry, University of California, Riverside, California, USA; 2 Environmental Toxicology Graduate Program, University of California, Riverside, California, USA

**Keywords:** epitranscriptomics, *N*^6^-methyladenosine, proteomics, RNA-binding proteins, RNA modifications

## Abstract

Epitranscriptomics is a rapidly evolving field that explores chemical modifications in RNA and how they contribute to dynamic and reversible regulations of gene expression. These modifications, for example, *N*^6^-methyladenosine (m^6^A), are crucial in various RNA metabolic processes, including splicing, stability, subcellular localization, and translation efficiency of mRNAs. Mass spectrometry-based proteomics has become an indispensable tool in unraveling the complexities of epitranscriptomics, offering high-throughput, precise protein identification, and accurate quantification of differential protein expression. Over the past two decades, advances in mass spectrometry, including the improvement of high-resolution mass spectrometers and innovative sample preparation methods, have allowed researchers to perform in-depth analyses of epitranscriptomic regulations. This review focuses on the applications of bottom-up proteomics in the field of epitranscriptomics, particularly in identifying and quantifying epitranscriptomic reader, writer, and eraser (RWE) proteins and in characterizing their functions, posttranslational modifications, and interactions with other proteins. Together, by leveraging modern proteomics, researchers can gain deep insights into the intricate regulatory networks of RNA modifications, advancing fundamental biology, and fostering potential therapeutic applications.

## 1 | Introduction

DNA undergoes dynamic chemical modifications to regulate gene expression, where DNA (cytosine-5)-methyltransferases catalyze the formation of 5-methylcytosine (5mC) in gene promoters to silence gene expression (Du, Wang, and Schramm 2016; Moore, Le, and Fan 2013). Cytosine methylation, together with histone posttranslational modifications (PTMs) and small noncoding RNAs (ncRNAs), allows for dynamic modulation of gene expression in response to cellular needs, for example, proliferation and differentiation (Liu and Pan 2016; Zhang, Fu, and Zhou 2019). Such gene expression regulation without altering DNA sequence is termed “epigenetics” and has been extensively investigated (Felsenfeld 2014).

RNA modifications, for example, pseudouridine (ψ), were identified at around the same time as DNA methylation (Cantara et al. 2011). However, the field only emerged after the availabilities of modification-specific sequencing techniques and the discovery of modification removal enzymes such as fat mass and obesity-associated (FTO) protein that demethylates *N*^6^-methyladenosine (m^6^A) (Linder et al. 2015; Liu et al. 2020; Merkestein et al. 2015). Viewing that posttranscriptional modifications are also involved in regulating gene expression without altering RNA sequence, this type of regulation is consequently coined as “epitranscriptomics” (He 2010).

In mammalian cells, transfer RNAs (tRNAs), which are adapter molecules bringing amino acids to the messenger RNA (mRNA) and pair with the corresponding codons through their anticodons during protein synthesis (Holley et al. 1965), are the most extensively modified RNAs in cells (Helm and Alfonzo 2014; Suzuki 2021). The modifications in anticodon regions are crucial for tRNAs to base pair with mRNA, thereby promoting reading frame maintenance and translation efficiency. In addition, those modifications occurring on tRNA body regulate their stabilities (Han and Phizicky 2018). Moreover, posttranscriptional modifications in ribosomal RNA (rRNA) regulate the translational efficiencies of mRNAs (Sloan et al. 2017).

In mRNA, *N*7-methylguanosine (m^7^G) is installed on the first nucleotide (Ramanathan, Robb, and Chan 2016). The resulting “mRNA cap” is crucial for posttranscriptional processing of mRNAs, including polyadenylation, splicing, and nuclear export (Lewis and Izaurflde 1997). In the cytosol, the m^7^G cap is involved in recruiting translation initiation factors for protein synthesis, thereby regulating translation efficiency (Andreev et al. 2009). Additionally, the cap m^7^G prevents mRNAs from exonuclease cleavage, suggesting its crucial role in promoting mRNA stability (Furuichi, LaFiandra, and Shatkin 1977). Aside from the constitutively installed m^7^G, m^6^A is the most abundant internal modification in mRNAs (Meyer and Jaffrey 2014). Recently, m^6^A was identified as a reversible modification regulating various aspects of RNA metabolism. Furthermore, m^6^A facilitates rapid and dynamic regulation of gene expression in response to extracellular stimuli and intracellular cues, which is achieved through its reader, writer, and eraser (RWE) proteins (Meyer and Jaffrey 2014).

Mass spectrometry (MS)-based proteomics constitutes a powerful tool for investigating systematically the proteome with high throughput and identification/quantification accuracy (Kwon et al. 2021; Latterich, Abramovitz, and Leyland-Jones 2008). Advances in MS in the last two decades, including the development of high-resolution mass spectrometers, data acquisition methods, and data analysis tools, foster the discoveries of numerous important discoveries in biology, including epitranscriptomics (Orsburn 2021; Wong et al. 2009; Zubarev and Makarov 2013). Moreover, some proteomics-driven findings in epitranscriptomics were translated into therapeutic developments (Chen et al. 2023). In modern MS-based proteomics, the top-down approach, which analyzes intact proteins directly by MS, provides comprehensive and specific molecular information. On the other hand, bottom-up proteomics, which interrogates proteolytically cleaved peptides, is the most widely used technique and has been extensively employed for investigating epitranscriptomic regulations.

In this review, we explore the applications of bottom-up proteomics techniques in the field of epitranscriptomics, focusing on the identification and quantification of epitranscriptomic RWE proteins. We also review the use of proteomics in interrogating the functions, PTMs, and protein-protein interactions (PPIs) of these RWE proteins.

## 2 | Epitranscriptomic Regulations

RNA is known to harbor more than 100 distinct types of chemical modifications. Among them, m^6^A is the most abundant internal modification in mRNAs and is the best-studied epitranscriptomic modification. In this section, we briefly review the cellular protein machinery involved in the recognition, installation, and removal of this modified nucleoside in RNA.

### 2.1 | m^6^A Installation and Removal

**FIGURE 1 |:**
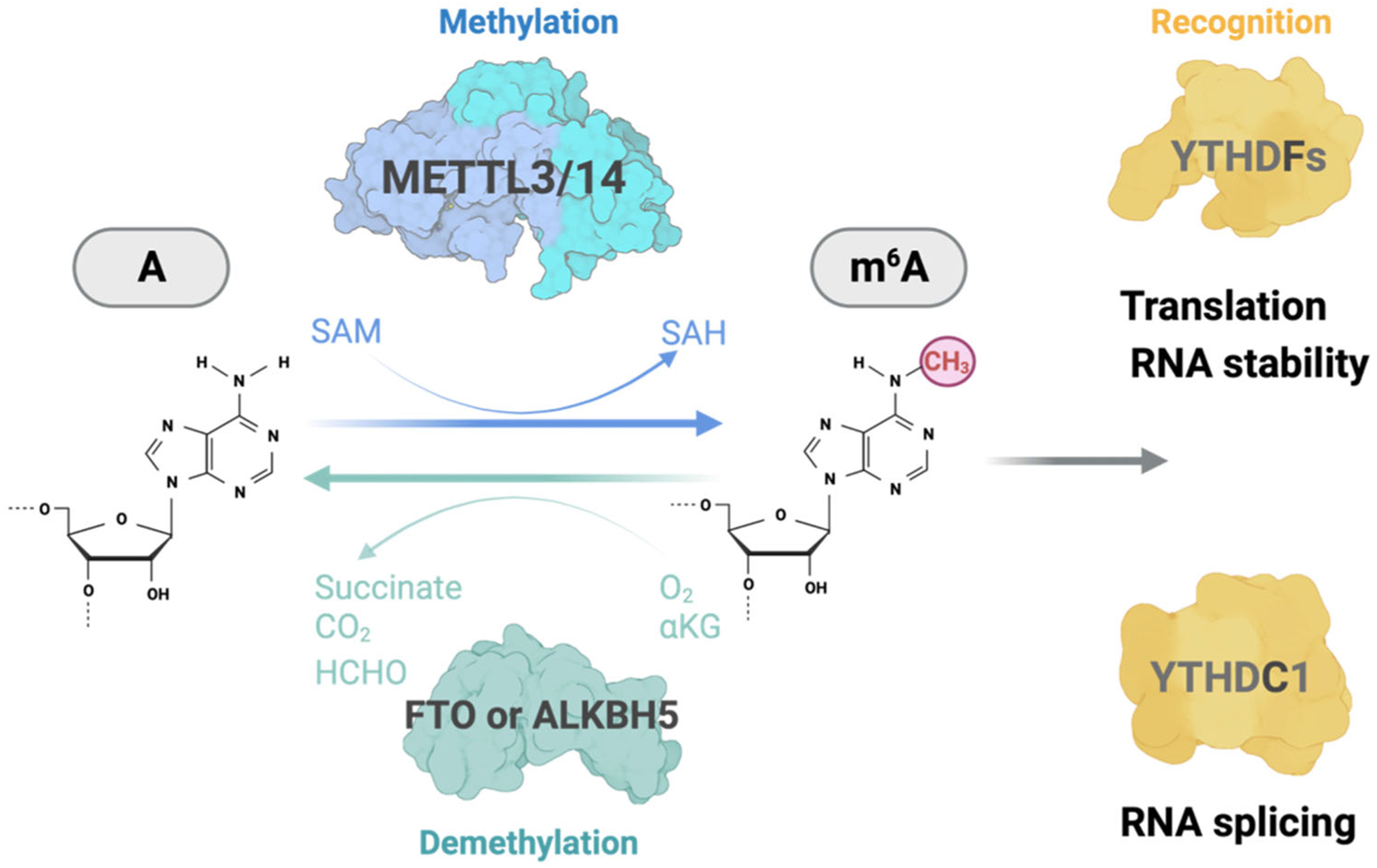
A schematic diagram showing m^6^A methylation in RNA. The adenosine at the center of GAC or AAC motif could be recognized by the METTL3/METTL14 methyltransferase complex to form m^6^A modification. The methylation could be removed by demethylases, including FTO and ALKBH5. The m^6^A-modified RNA could be recognized by reader proteins to further modulate RNA metabolisms such as mRNA stability and translational efficiency.

In mammalian cells, m^6^A is installed in a consensus sequence motif of GAC or AAC in mRNAs and ncRNAs with the central A being methylated. In the current paradigm, the methylation is installed by a methyltransferase complex centered by the methyltransferase-like 3 (METTL3)/methyltransferase-like 14 (METTL14) heterodimer. In this vein, METTL14 in the complex does not exhibit any catalytic activity, instead it assumes a structural role in enabling METTL3 to recognize RNA substrates (Figure 1) (Wang, Doxtader, and Nam 2016; Wang, Feng, et al. 2016).

Aside from the METTL3/METTL14 heterodimer, METTL16 was shown to install m^6^A on the MAT2A mRNA, which encodes *S*-adenosyl-l-methionine (SAM) synthetase, and U6 snRNA on a consensus sequence motif of CAG. The crystal structure of METTL16 in complex with MAT2A mRNA revealed that the enzyme recognizes mRNA loop through its N-terminal domain, suggesting that the consensus sequence alone is insufficient for METTL16 recognition, but instead the methylation entails specific secondary structure of RNA. Pendleton et al. (2017) demonstrated that, under low SAM concentration, METTL16 retains on the 3’-untranslated region (3’-UTR) of MAT2A mRNA. Interestingly, such retention of METTL16 on the MAT2A transcript promotes its splicing and subsequent maturation to allow for cells to synthesize more SAM. Given that SAM is the predominant source of methyl group for most methylations in cells, including those of DNA, RNA, and proteins, it is not surprising that METTL16 depletion pronouncedly disrupts cellular regulations. Mendel et al. (2018) later showed that genetic ablation of *Mettl16* led to drastic dysregulation in the transcriptome of mouse embryos and impaired embryonic development starting at the ~64-cell blastocyst stage. Thus, although METTL16 is currently known to regulate only a few mRNA targets, the methyltransferase is indispensable for cell survival and development.

To achieve dynamic regulations, m^6^A could be removed by demethylases in cells (Figure 1). At around the same time when the METTL3-METTL14 methyltransferase was discovered, FTO was found to demethylate m^6^A in vitro and in vivo (Jia et al. 2011). In addition, FTO was identified as a nuclear m^6^A demethylase that is localized in nuclear speckles to process certain newly transcribed small ncRNAs and pre-mRNAs (Meyer and Jaffrey 2014). Other studies documented that FTO may also localize in the cytosol in certain neurons, suggesting that the functions of this demethylase might be cell-type-dependent (Hess et al. 2013).

Another identified m^6^A demethylase is the α-ketoglutarate-dependent dioxygenase alkB homologue 5 (ALKBH5), an ortholog of FTO. Similar to FTO, ALKBH5 is also localized in nuclear speckles to regulate mRNA processing. The results further suggest that ALKBH5 could remove m^6^A to regulate mRNA transport. mRNAs were found to accumulate in the nucleus in ALKBH5-deficient cells, and *Alkbh5^−/−^* mice exhibit defective spermatogenesis (Zheng et al. 2013).

### 2.2 | m^6^A Reader Proteins and Cellular Functions

The functions of m^6^A in mRNAs are realized through its recognition by cellular proteins. The methylation may also alter the structures of mRNAs to modulate their interactions with proteins. Liu et al. (2015) demonstrated that m^6^A alters the local structure of long ncRNAs to promote their binding with heterogeneous nuclear ribonucleoprotein C (hnRNPC). Notably, hnRNPC does not directly recognize m^6^A; instead, the binding is modulated by the m^6^A-elicited change in RNA structure. The resulting mRNA–hnRNPC interaction modulates mRNA splicing (Liu et al. 2015).

YTH domain-containing 1 (YTHDC1) was identified as the major nuclear m^6^A reader protein. Upon binding to m^6^A-containing RNA, YTHDC1 recruits SR family trans-acting splicing factors (e.g., SRSF3) to process pre-mRNAs, and to promote exon inclusion of target genes (Xiao et al. 2016). Recently, YTHDC1 was also shown to bridge epitranscriptomic and epigenetic regulations. For instance, Xu et al. (2021) discovered that, in mouse embryonic stem cells (mESCs), YTHDC1 recognizes METTL3-deposited m^6^A on chromatin-bound IAPE-z ncRNA. In addition, YTHDC1 interacts with and recruits METTL3 to chromatin, which leads to elevated chromatin occupancy of the SETDB1–TRIM28 H3K9me3 methyltransferase complex. Hence, the YTHDC1–METTL3 interaction amplifies the signal of m^6^A in chromatin-associated RNA and induces hypermethylation of histone H3K9 to form heterochromatin. In addition, Li et al. (2020) showed that the repressive histone mark H3K9me2 could be removed by YTHDC1-recruited lysine-specific demethylase 3B, thereby stimulating gene expression in differentiated cells.

In the cytosol, m^6^A is recognized by the YTH domain family proteins, including YTHDF1, YTHDF2, and YTHDF3, where the interactions regulate RNA metabolisms (Wang et al. 2014). The mRNAs, when bound with YTHDF2, are susceptible to degradation. Wang et al. (2014) demonstrated that YTHDF2 recognizes m^6^A-modified RNA through its C-terminal domain, and depletion of YTHDF2 led to increased lifetimes of its target transcripts in cells. Du, Zhao, et al. (2016) further explored the mechanism through which YTHDF2 binding promotes mRNA decay. In particular, they found that the N-terminal region of YTHDF2 interacts directly with the SH domain of the CNOT1 within the CCR4–NOT complex, thereby recruiting the complex to deadenylate YTHDF2-bound m^6^A-containing mRNAs and promoting their decay (Du, Zhao, et al. 2016).

Apart from RNA destabilization, m^6^A in mRNA is also involved in regulating translation efficiency. Meyer et al. (2015) demonstrated that the m^6^A modification in the 5’-UTR could stimulate cap-independent translation. The authors further showed that eukaryotic initiation factor 3 (eIF3) binds to m^6^A in the 5’-UTR of mRNAs, of which the mRNA–protein interaction is sufficient to recruit the ribosomal complex for translation initiation even without the aforementioned m^7^G cap. Additionally, Choe et al. (2018) showed that METTL3 installs m^6^A in the 3’-UTR, during which METTL3 physically interacts with translation initiation factor eIF3h to promote ribosome recycling via mRNA looping. Through the m^6^A-dependent PPIs, the 3’-UTR, where the ribosome complex falls off after translation, is brought into close proximity of the 5’-UTR, allowing for augmented ribosome occupancy at the translation initiation sites.

Together, m^6^A regulates a wide spectrum of RNA metabolisms. In the nucleus, m^6^A is recognized by the reader protein YTHDC1 to modulate histone epigenetic marks, thereby regulating gene expression. During posttranscriptional processing, m^6^A could recruit splicing factors or alter local RNA structure to modulate RNA–protein interactions. In the cytosol, m^6^A-modified mRNAs could be recognized by YTHDF family proteins to regulate the stability and translation efficiency of mRNAs. Future studies about the molecular mechanisms will bring additional insights into the functions of m^6^A and other modified nucleosides in RNA biology.

## 3 | MS-Based Quantitative Proteomics

**FIGURE 2 |:**
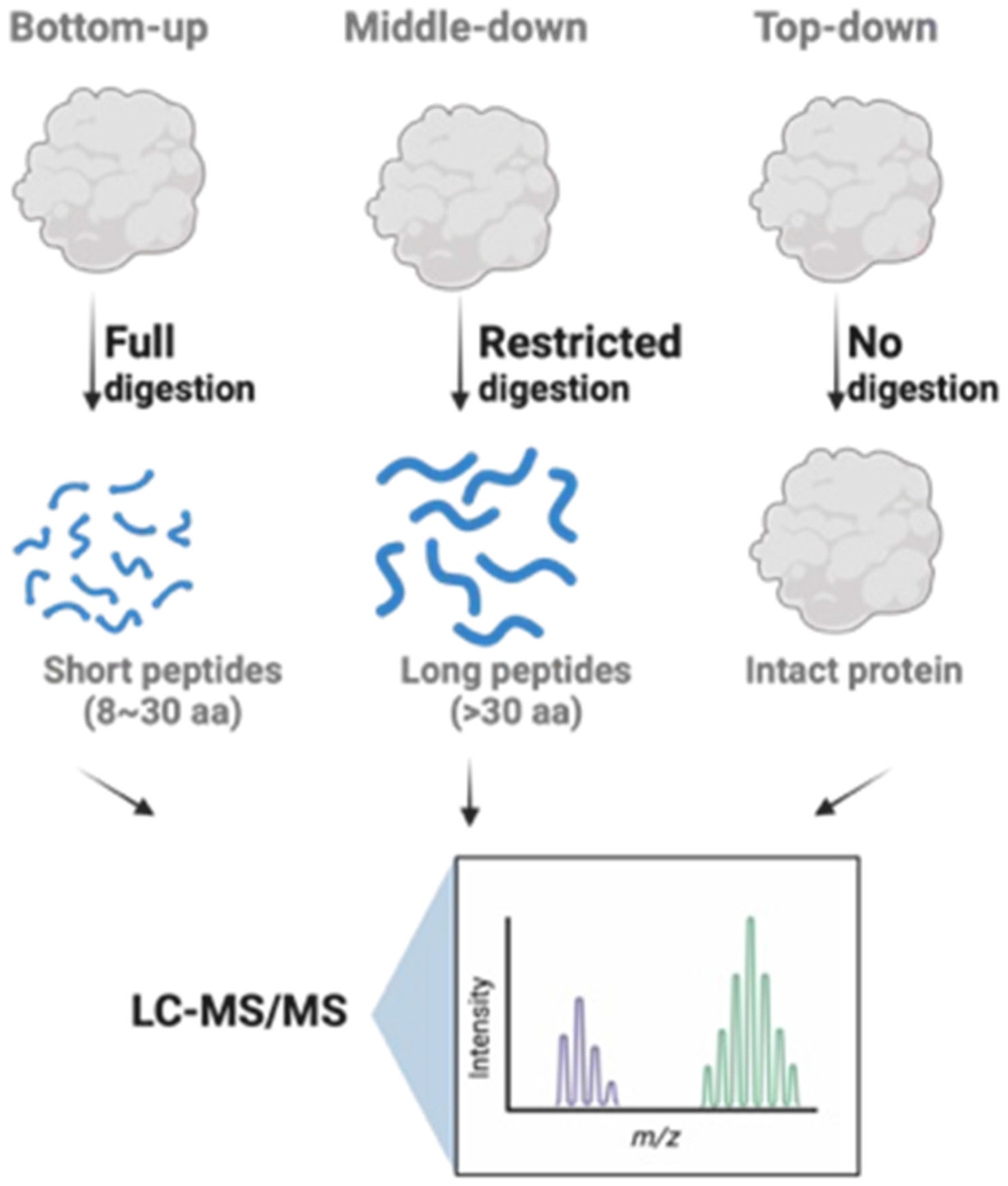
Overview of the workflows for common proteomics approaches, including bottom-up, middle-down, and top-down.

While RNA sequencing has been employed as a surrogate to quantify gene expression, the results do not always align with those of protein expression (Liu, Beyer, and Aebersold 2016). Translational efficiency and protein turnover both contribute to the dynamic regulation of protein expression on top of its mRNA expression. Moreover, proteins can be chemically modified to exert their functions in the cell. Thus, quantitative proteomics could offer important insights into biological regulations. To date, various proteomic approaches have been developed to quantify protein expression using MS^1^ or MS^2^ scans (Figure 2). Among them, bottom-up proteomics is the most widely adopted and it involves enzymatic digestion of proteins into peptides before MS analysis. This method enables the identification and quantification of thousands of proteins in complex biological samples in high throughput.

Top-down proteomics analyzes intact proteins rather than their digested peptide products, allowing for characterizing full-length proteins and their chemical modifications (Moradian et al. 2014; Po and Eyers 2023). The results from top-down proteomics enable systematic investigation of epitranscriptomic RWE proteins, including protein isoforms, of which the information is often missed in bottom-up approach. Moreover, top-down proteomics can identify combinatorial PTM sites and protein–substrate interactions of specific proteoforms, offering detailed insights into the regulation of the epitranscriptomic RWE proteins.

Middle-down proteomics, in contrast, bridges the gap between top-down and bottom-up approaches. In the middle-down approach, proteins are partially cleaved into larger peptide fragments rather than fully digested peptides, allowing for the preservation of functional context, such as the combination of PTMs (Sidoli and Garcia 2017). In this regard, middle-down proteomics can be used to examine how specific domains of epitranscriptomic RWE proteins are modified, and how these modifications influence protein complex formation and function. However, despite the potential applications of top-down and middle-down proteomics in epitranscriptomic research, to date the regulations of epitranscriptomic RWE proteins have predominantly been characterized using bottom-up proteomics owing to its higher throughput and wider availability. Therefore, the primary focus of this review will be placed on the applications of bottom-up proteomics for examining the regulations of epitranscriptomic RWE proteins.

### 3.1 | MS^1^-Based Quantification

#### 3.1.1 | SILAC

Recent developments in MS-based proteomics enable automated analysis of thousands of proteins in a single LC-MS/MS run. Different labeling approaches have been developed for quantifying protein expression. Among them, SILAC, which was introduced by Ong et al. (2002) in the early 2000s, has been extensively employed in quantitative proteomics due to its quantification accuracy. In SILAC experiments, the cells are cultured in media containing unlabeled (light) or stable isotope-labeled (SIL) (heavy) essential amino acids, which could not be synthesized in mammalian cells (Geiger et al. 2010; Ong et al. 2002). Thus, the only source of these amino acids is from the culture medium, which facilitates their incorporation into the proteomes of cultured cells.

Currently, a combination of proteolysis with trypsin, a protease that cleaves amide bonds on the C-terminal side of lysine and arginine, and SILAC labeling with ^13^C- and ^15^N-labeled lysine and arginine is the most commonly used (Ong et al. 2002). In this combination, all tryptic peptides except the C-terminal peptides of some proteins harbor at least one heavy isotope-labeled amino acids, allowing for comprehensive and unbiased peptide quantification. Notably, arginine is actually not characterized as an essential amino acid; however, its inclusion in the culture media is required for normal proliferation of many cell lines (Wheatley et al. 2000). Arginine was shown to be converted to proline in some cell lines (e.g., HeLa cells) (Bendall et al. 2008). This conversion may complicate data analysis and, if not incorporated properly into the data analysis workflow, distorts the quantification results. To resolve this issue, one can supplement the SILAC medium with excess unlabeled proline to suppress the conversion (Bendall et al. 2008; Lößner et al. 2011).

For successful SILAC-based protein quantification, complete incorporation of the heavy isotope-labeled amino acids into the proteome is essential. To achieve this, the cells must be cultured in SILAC medium for sufficient time depending on the cell doubling time, and the incorporation rate must be examined up-front through LC-MS/MS analysis (Ong and Mann 2006). To prevent biased quantification results arising from incomplete incorporation of heavy isotope-labeled amino acids, the assignments of SILAC media to the testing samples are often swapped in different biological replicates. Immediately after harvesting, cells cultured in different SILAC media are combined by either equal cell number or protein quantity, and the ensuing mixture is subjected to protease digestion. Given that all samples are combined at the very beginning, variations in subsequent steps of sample preparation do not compromise quantification accuracy. Thus, SILAC is a straightforward and highly accurate quantitative proteomics approach. In addition, the light and heavy forms of each peptide, due to their identical amino acid sequence, exhibit nearly the same elution time in LC. Consequently, interferences emanating from different instrument conditions, for example, ionization efficiency and sample matrices, bear negligible effects on relative peptide quantification. During LC-MS/MS analysis, each peptide appears as a pair in both MS^1^ and MS^2^, with the quantification being predominantly achieved using the chromatographic peak area of the former.

#### 3.1.2 | Nonisobaric Chemical Labeling

SILAC labeling procedures, despite being successfully adapted to higher model organisms (e.g., mice) (Rayavarapu et al. 2013), are inherently very involved. Consequently, the approach is not cost-effective for most projects that include animal studies or clinical samples. The development of the super-SILAC approach (Geiger et al. 2010), which involves mixing heavy protein lysates from multiple cell lines, partially resolves the issue, yet proper selection and evaluation of cell lines could be time-consuming and may not encompass all proteins present in the samples to be analyzed. The nonisobaric labeling for peptide quantification using MS^1^ was first realized through the development of an isotope-coded affinity tag (ICAT) in the late 1990s (Gygi et al. 1999; Sethuraman et al. 2004). The approach involves reaction of free cysteine residues in proteins with thiol-specific, deuterium-labeled, and biotinylated iodoacetamide probe, and the labeled peptides are subsequently enriched through affinity purification and analyzed by LC-MS/MS. However, due to the relatively low frequency of occurrence of cysteines in proteins, it is estimated that only 10%–20% of the whole-cell proteome could be quantified using ICAT, which left a large fraction of the proteome uncharacterized. One way to overcome this limitation is to label peptides through their free amines (N-terminus and the lysine side chain).

Reductive dimethyl labeling, which was pioneered by Hsu et al. (2003) and further optimized by others (Boersema et al. 2008, 2009), is an efficient and inexpressive approach for converting primary amines to dimethylamines with different combinations of ^13^C and deuterium-labeled reagents. Recently, mass difference tandem mass tagging (mTMT) (Paulo and Gygi 2019) and tag for relative and absolute quantitation (mTRAQ) (Oppermann et al. 2013) that carry amine-reactive NHS-ester moiety were developed as alternative approaches. Unlike dimethyl labeling that may suffer from retention time drift due to the use of deuterium, mTMT, and mTRAQ, which employ ^13^C and ^15^N stable isotopes, allow for the coelution of the labeled peptides. Thus, the quantification is not affected by fluctuation in chromatography conditions. Nonisobaric stable isotope labeling is an attractive approach for MS^1^-based quantitative proteomics. In addition, the approach provides improved quantification precision and accuracy, particularly in targeted proteomics (Yin et al. 2013). Furthermore, Oppermann et al. (2013) showed that SILAC- and mTRAQ-based approaches exhibit comparable quantification results in phosphoproteomics analysis, highlighting nonisobaric stable isotope labeling as a useful alternative to metabolic isotope labeling. Together, nonisobaric stable isotope labeling is a robust alternative to SILAC.

#### 3.1.3 | Label-Free Quantification

Over the years, MS-based quantitative proteomics becomes indispensable in investigating biological regulations. While the aforementioned labeling approaches have been developed for relative quantification, they are not always applicable due to the nature of the samples. For instance, although SILAC provides high accuracy, generally metabolic labeling procedures cannot be transferred to clinical studies. The aforementioned non-isobaric labeling approach introduces extra steps that result in additional sample loss, thus prevents its applications for low-input samples. In this regard, label-free quantification, which requires no additional chemical labeling or sample preparation, is gaining popularity in fields like clinical proteomics.

Unlike the labeling-based MS^1^ quantification approaches that combine multiple samples for a single LC-MS/MS analysis, label-free quantification simply involves analyzing these samples separately by LC-MS/MS. Label-free quantification is subsequently achieved through comparing peak areas in the extracted-ion chromatograms of precursor ions in most scenarios. Chelius and Bondarenko (2002) demonstrated that the peak area correlates linearly with peptide concentration in samples, laying the foundation for chromatogram-based label-free quantification. However, caution should be exerted about the dynamic range of the mass analyzer to prevent over- or underestimating the relative quantification results.

Alternatively, MS^2^ spectra counting has been used as a surrogate for relative peptide abundance (Liu, Sadygov, and Yates 2004). In this approach, the number of MS^2^ spectra of a given peptide are summed as the spectral count. To quantify a protein, the spectral count of all the peptides derived from the protein of interest is compared across samples. Data processing approaches have been developed to unbiasedly convert the differential spectral counts into relative protein expression (Griffin et al. 2010). However, it has been shown that the method still performs poorly in terms of quantification accuracy. Additionally, spectral counting approach requires high spectrum scanning speed, which is not always achievable. Moreover, the required recurrent MS^2^ acquisition conflicts with dynamic exclusion that is often implemented to prevent repetitive precursor ion selection. Besides, the approach has limited dynamic range, further hindering its application in quantifying protein expression. Thus, spectral counting approach currently remains controversial (Bantscheff et al. 2007).

The accuracy of label-free quantification has been significantly improved owing to the recent developments in computational proteomics (Arike et al. 2012; Cox et al. 2014; Schwanhäusser et al. 2011). A recent extensive assessment of label-free quantification software showed that tools like MaxQuant or Progenesis QIP can accurately quantify those proteins with abundances varying by two orders of magnitude (Al Shweiki et al. 2017). Another advantage of label-free quantification is the deep proteome coverage. In the nonisobaric labeling-based approaches, the same peptide needs to be scanned at least twice, which consumes additional instrument time to acquire the quantification data of the same peptide.

A common drawback of label-free approach in data-dependent acquisition (DDA) analysis is the reproducibility in identification and quantification results, which arises from different instrumental conditions and the stoichiometry of peptides. Hence, missing data, where a protein is only detected in a subset of samples, is frequently encountered. Such absence of protein signal intensity in some samples complicates data interpretation. To avoid missing data, label-free quantitation in data-independent acquisition (DIA) mode has been developed (Röst et al. 2014; Yu et al. 2023; Zhang et al. 2020). In addition, Shen et al. (2018) developed the IonStar approach, which utilizes MS^1^-based quantifications. The approach demonstrated nearly complete identification in large cohort studies, underscoring the method’s sensitivity and reproducibility. Together, because of its simplicity and sample compatibility, label-free quantification is a promising technique in quantitative proteomics. A detailed discussion of label-free quantification can be found elsewhere (Li et al. 2012; Neilson et al. 2011).

### 3.2 | MS^n^-Based Quantification–Isobaric Labeling

**FIGURE 3 |:**
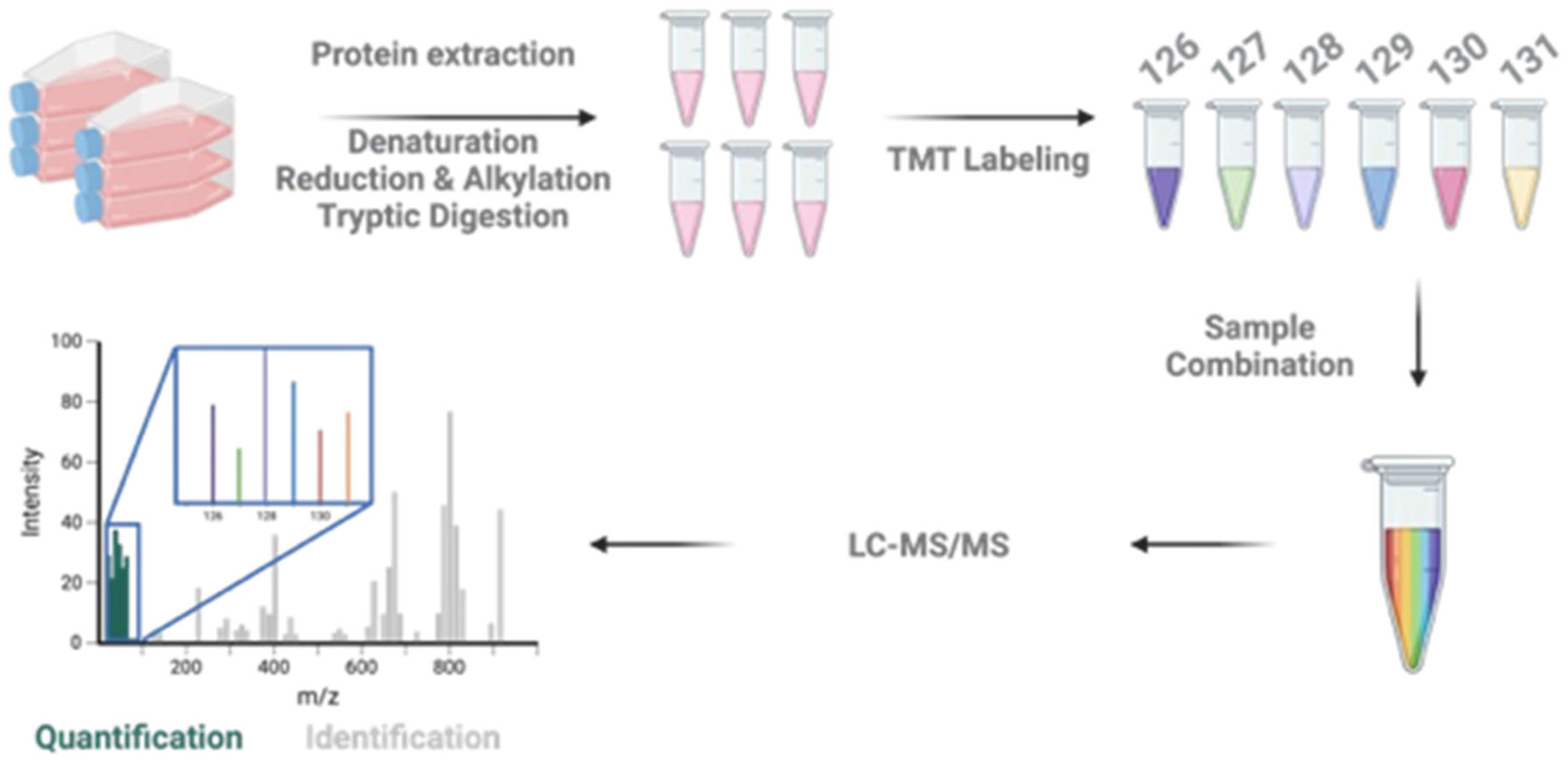
A schematic diagram illustrating the workflow of TMT-based quantitative proteomics. Proteins extracted from cells are digested by trypsin, and the resulting peptides are labeled by 6-plex TMT reagents. Precursor ions of peptides are isolated for fragmentation. The low-mass reporter ions are monitored in MS^2^ scan for quantification.

Large-scale quantitative proteomics is powerful in investigating systematically biological regulations directly at the protein level. To improve data acquisition rate and allow for simultaneous analysis of multiple samples on mass spectrometers, isobaric mass tags, which label peptides after protease digestion, have been developed (Figure 3). The isobaric mass tag is comprised of a reactive group to react with peptides, a reporter group linked by an isotopic mass balancer group, of which the bond is susceptible to cleavage during collisional activation. The nominal mass of each of the isobaric mass tags is identical while the stable heavy isotopes (^13^C, ^15^N, or ^18^O) are distributed differently between the reporter and balancer groups (Figure 3). In this regard, isobarically labeled peptides share almost identical LC elution profile and are indistinguishable in the MS^1^ scan. During fragmentation, reporter ions are released, and the balancer group is lost as a neutral molecule. The peak intensities of the ensuing reporter ions are used for relative quantification. It is noteworthy that the reporter ions reside in the low *m/z* range; hence, they do not interfere with the sequence-informative *b*- and *y*-fragment ions distributed in higher *m/z* range.

Both TMT and isobaric tags for relative and absolute quantitation (iTRAQ) are commonly used in isobaric labeling. In the early 2000s, Thompson et al. (2003) introduced, for the first time, TMT strategy for achieving relative quantification. While only 2-plex was available at the time, the multiplexing capacity has been increased to 16-plex to date, with 6- and 10-plex being the most widely used (Li et al. 2020). In TMT 6-plex, the reporter ions generated are of *m/z* 126, 127, 128, 129, 130, and 131 (Dayon et al. 2008). The 6-plex was later expanded to 10-plex without altering the chemical structure of the tag (McAlister et al. 2012). Instead, the isotopic mass shift of reporter ions introduced by ^13^C differs from that of ^15^N by 6.32mDa, which could be resolved by high-resolution mass spectrometers like Orbitrap to achieve relative quantification. Recently, Wang et al. (2020) demonstrated that combining TMT reagents of different chemical structures, which result in mass shifts of precursor ions, allows for the MS^1^-based quantification. Using this approach, the multiplexing capacity could be further increased to 27-plex, underscoring that the throughput could be further enhanced by combining MS^1^- and MS^2^-based approaches.

iTRAQ was introduced by Ross et al. (2004) as a 4-plex shortly after the development of TMT. In 4-plex iTRAQ, the reporter ions are at *m/z* 114, 115, 116, 117, which were later expanded to 8-plex with reporter ions at *m/z* from 113 to 119 and 121 (Choe et al. 2007). Notably, the reporter ion of *m/z* 120, which is nearly identical to the *m/z* value of the immonium ion of phenylalanine, was omitted to prevent distorting the quantification results. Other isobaric labeling techniques include *N,N*-dimethyl leucine (DiLeu) (Frost, Greer, and Li 2015; Xiang et al. 2010) and deuterium isobaric amine-reactive tag (DiART) (Zhang, Wang, and Li 2010).

An advantage of using isobaric labeling as compared to metabolic labeling such as SILAC is that the samples are combined for a single injection, where the labeled peptides co-elute and appear as a single peak in MS^1^, allowing for enhanced signal intensity. While metabolic labeling has been applied to animal studies, the technique is predominantly suitable only for cultured cells. Isobaric labeling, similar to the aforementioned nonisobaric labeling, is immune to this restriction and has been extensively used in clinical proteomics (Dayon et al. 2008; Wang et al. 2020). Another advantage of using isobaric labeling is that the incidence of missing values is typically very rare. Given that missing quantification information complicates downstream data processing and often requires imputations for statistical analysis, isobaric labeling allows for more straightforward data interpretation. A recent study demonstrated that more than 99% of proteins are commonly quantified within the same TMT batch with the coefficient of variation (CV) being threefold lower than that of label-free quantification (O’Connell et al. 2018). Another study showed that approximately 90% of proteins are commonly quantified even in the 24-batch experiment of 10-plex TMT-labeled samples, highlighting the robustness of the isobaric labeling approach in reducing missing values (Brenes et al. 2019). Moreover, Gygi et al. (1999) and Nusinow et al. (2020) interrogated the proteome of 375 cell lines in the Cancer Cell Line Encyclopedia (CCLE), of which the samples were consolidated into only 42 runs by TMT-based isobaric labeling, underscoring the excellent throughput of the method.

A well-known limitation of isobaric labeling is ratio compression, where the co-eluting and nearly isobaric peptides contaminate the target peptides of interest, leading to distorted quantification results. To address this issue, McAlister et al. (2014) developed an MS^3^ method using isolation waveforms with multiple frequency notches, which allows for isolation of multiple MS^2^ fragment ions for further fragmentation and enables quantification using reporter ions detected in MS^3^. Because it is unlikely for the contaminating and desired precursor ions to generate identical fragment ions in MS^2^, the approach allows for the reporter ions to be derived exclusively from the targeted peptide. An alternative approach to overcome the ratio compression involves imposing a narrower *m/z* window for precursor ion isolation (Savitski et al. 2011). Another drawback of isobaric labeling is that it is generally not suitable for targeted proteomics. This is due to the fact that the labeled peptides could not be distinguished in MS^1^, which loses the two-step selection advantage in multiple-reaction monitoring (MRM) or parallel-reaction monitoring (PRM) to achieve superior selectivity. In-depth discussion about different isobaric labeling approaches can be found elsewhere (Rauniyar and Yates 2014).

Large-scale quantitative proteomics is becoming indispensable for gaining insights into epitranscriptomic regulations directly at the protein level rather than using mRNA expression as a surrogate. Such projects sometimes involve the analyses of hundreds of samples. Thus, isobaric labeling enables multiplexing in LC-MS/MS analysis and allows for acquiring results in a timely and high-throughput manner. We expect that it will be widely employed in large-scale quantitative proteomics studies of epitranscriptomic regulations.

## 4 | Identification and Quantifications of Epitranscriptomic RWE Proteins

### 4.1 | MS-Assisted Discovery of Epitranscriptomic Writer Complexes

Proteins are seldom solitary agents within cellular environments; it is estimated that over 80% of proteins, including those involved in epitranscriptomic regulations form complexes to achieve precise spatiotemporal regulation. The compositions of these complexes are dynamically regulated by various factors such as PPIs and/or PTMs (Liu et al. 2014). In cells, METTL3, a key epitranscriptomic writer protein, forms a heterodimer with METTL14, creating a positively charged groove essential for RNA binding (Wang, Doxtader, and Nam 2016). This structural configuration enables METTL3 to effectively bind to the methyl group donor, that is, SAM, and catalyze the deposition of m^6^A on RNA. This interaction not only illustrates the cooperative nature of protein complexes in modulating RNA but also highlights the sophisticated regulatory mechanisms governing epitranscriptomic modifications, which are critical for a myriad of cellular processes.

Many key proteins involved in m^6^A methylation were initially identified through conventional biochemical methods; however, the mechanistic intricacies of RNA methylation were elucidated only after deeper exploration using co-immunoprecipitation (co-IP) coupled with MS-based proteomics. METTL3, a crucial catalytic component of the major m^6^A methyltransferase complex, was first discovered by Bokar and colleagues (Bokar et al. 1994) through nuclear protein fractionation. After that discovery, it took nearly two decades for a detailed study of the METTL3-centered methyltransferase complex due to limited knowledge in epitranscriptomics at the time. Advances in techniques such as high-throughput RNA sequencing and high-resolution mass spectrometers enabled researchers to investigate epitranscriptomic regulations since the initial discovery of METTL3 (Liu et al. 2014). Following these pioneering discoveries facilitated by MS, interest in RNA modifications gained significant momentum in the early 2010s, leading to rapid development of the epitranscriptomics field.

In co-IP experiments, an antibody is immobilized or captured by protein A/G beads to recognize and subsequently purify a member of the epitranscriptomic protein complex from lysates. In that process, the antibody pulls down the targeted epitranscriptomic regulatory protein, with which the associated proteins that bind directly or indirectly are co-enriched. Following adequate washing, proteins could be digested on beads or eluted for standard proteomic sample preparation workflow. Through proteomics analysis, the interaction network of the targeted protein could be delineated.

Successful enrichment with minimal background is essential in co-IP experiments. In this regard, antibodies with high specificity and affinity to the epitranscriptomic regulatory protein of interest must be carefully chosen and tested up-front. Alternatively, tags such as Flag could be genetically incorporated to be expressed in-frame with the protein of interest (Berggård, Linse, and James 2007). Recently, advances in tagging technology, such as the introduction of Halo tag offer increased specificity (England, Luo, and Cai 2015). Given that commercial antibodies for the frequently used tags are often of high quality, tagging approach typically allows for clean and effective enrichment. This technique provides a robust tool for elucidating complex protein networks and their dynamics in cellular processes, offering insights into the molecular mechanisms driving various epitranscriptomic regulations.

Liu et al. (2014) advanced the understanding of the METTL3 methyltransferase complex by showing that METTL14 and WTAP are crucial components of the complex. In particular, their LC-MS/MS analyses of Flag-tagged METTL3, METTL14, and WTAP individually pulled down from HeLa cell lysates revealed the presence of the other two proteins. Simultaneously, Ping et al. (2014), by using co-IP on SFB-tagged METTL3 from 293T cells, confirmed that the methyltransferase complex includes METTL3, METTL14, and WTAP. The MS results from these studies unambiguously confirmed the core components of the methyltransferase complex, paving the way for subsequent ground-breaking findings in the field of epitranscriptomics. These studies highlight the critical role of advanced proteomic techniques in uncovering the molecular basis of m^6^A and its regulatory mechanisms.

Beyond the basic methyltransferase complex, writer proteins such as METTL3 often form additional unique complexes to facilitate specific regulatory functions in different cellular compartments. MS is particularly well-suited for discovering these regulation-specific complexes owing to its high throughput, sensitivity, and specificity. An illustrative case is provided by the work of Choe et al. (2018), who purified Flag-tagged METTL3 protein from cells for MS analyses. They successfully identified several translation factors that exist in the same protein complex as METTL3. Notably, the interaction between METTL3 and specific translation factors, for example, eIF3, was subsequently found to enhance translation efficiency (Choe et al. 2018). This enhancement occurs through the tethering of the 5’- and 3’-UTRs of mRNA, as discussed above.

Apart from co-IP, proximity labeling has become a valuable method for examining PPIs with high spatiotemporal resolution, overcoming limitations of traditional co-IP experiments. Proximity labeling that typically employs an enzyme, for example, ascorbate peroxidase (APEX) or biotin ligase, fused with the protein of interest. These enzymes catalyze the biotinylation of nearby proteins, capturing a broad range of interactions, including ephemeral and spatially relevant contacts, without relying on physical stability through biochemical processing (Cho, Branon, Rajeev, et al. 2020; De Munter et al. 2017; Motani and Kosako 2020; Qin et al. 2021). This method enhances the detection of protein interactions, particularly beneficial to the discovery of epitranscriptomic complexes that are dynamic or held together through transient and/or weak PPIs (Hung et al. 2016). It is often desirable to deploy conditions, for example, urea-containing buffers, to eliminate effectively non-specifically bound proteins and ensure the isolation of specifically labeled proteins.

These findings underscore the importance of MS coupled with advanced sample preparation methods in elucidating novel epitranscriptomic regulatory proteins and in revealing their multifaceted roles in cellular processes. By identifying and characterizing these interactions, researchers can further unravel intricate regulatory mechanisms mediated by RNA modifications, opening up new venues for understanding epitranscriptomic regulations at the molecular level.

### 4.2 | Tips for Characterizing Epitranscriptomic Regulatory Protein Complexes by Using MS

#### 4.2.1 | Co-IP

Ideally, for the most accurate representation of native PPIs, the epitranscriptomic regulatory proteins should be enriched at their endogenous levels. This approach preserves the natural context and dynamics of their interactions. However, low expression levels of some target proteins may significantly diminish the efficiency of immunoprecipitation, leading to a reduction in the number of associated proteins that can be confidently identified. To overcome this limitation, a common strategy is to overexpress the protein of interest. This increase in protein abundance can enhance the efficiency of the pull-down process, rendering it easier to capture and identify interacting proteins. By engineering the target protein to include an epitope tag and using high-quality, antibody-conjugated beads specific to that tag, the co-IP process can be optimized. This method not only improves pull-down efficiency but also tends to be more reliable and specific, minimizing the capture of nonspecific binders and enhancing the overall data quality (Choe et al. 2018).

Since only a fraction of the bead’s surface is conjugated with antibodies, there is a risk that proteins could adsorb non-specifically to the unconjugated areas of the beads. To mitigate this, it is essential to sufficiently block these surfaces, where bovine serum albumin (BSA) is commonly used for this purpose. Considering the abilities of epitranscriptomic RWE proteins to bind RNA, it is advisable to block the beads with nucleic acids, for example, double-stranded DNA, to prevent RNAs from the lysate in binding to the beads. Those RNAs can attract numerous RNA-binding proteins, either relevant or irrelevant to the specific epitranscriptomic regulatory protein under investigation, and lead to false-positive discovery. Blocking beads with nucleic acids helps ensure that the proteins pulled down in the experiment are those genuinely associated with the targeted epitranscriptomic modulators, rather than nonspecific RNA-binding proteins (Cattoglio et al. 2020). Such meticulous attention to detail in blocking strategies greatly enhances the reliability of the data about PPIs of epitranscriptomic modulators. Removal of nucleic acids from cell lysate can further reduce the likelihood of nonspecific PPIs mediated through nucleic acids. The lysate should be treated with a nuclease, for example, benzonase, to digest nucleic acids, thereby ensuring that the PPIs are independent of nucleic acids. This step is crucial for ensuring that the interaction data reflect *bona fide* PPIs.

Incorporating proper controls into an experimental design is critical to avoid false-positive discoveries in co-IP experiments. This includes using samples from cells that do not express the tagged epitranscriptomic regulatory proteins of interest. These control experiments help define the baseline level of nonspecific binding in the system, providing a clear contrast to samples where the tagged protein is expressed and can interact with its potential partners. In addition, co-IP procedures require mild binding and washing conditions due to the use of antibodies. These conditions are crucial for preventing antibody denaturation and maintaining the native state and interactions of the proteins, but they can lead to high background noise in proteomics results. To effectively navigate the optimal co-IP conditions, conducting pilot experiments is essential. These preliminary tests help fine-tune the experimental conditions and ensure the robustness of the results. Here are some steps to consider in the evaluation: (1) Stability and preservation of known binders: It is helpful to utilize simpler and quicker methods, such as western blot analysis to verify if known interacting proteins are preserved through the co-IP process. This test can confirm whether the binding and washing conditions are stringent enough to reduce nonspecific interactions while preserving the integrity of protein complexes. (2) Quantitative evaluation of enriched proteins: It is crucial to assess the quantity of proteins in the IP sample relative to the input. Typically, the amount of proteins pulled down in a co-IP should represent a small fraction of the input, often less than 1%.

By carefully examining these aspects in pilot experiments, researchers can optimize the co-IP protocol to strike a balance between preserving protein interactions and reducing background noise. Such pilot experiments ensure that subsequent MS analyses yield specific and meaningful results, allowing for a clearer understanding of the protein interaction networks of interest.

#### 4.2.2 | Proximity Labeling

The aforementioned antibody-based affinity purification in combination with MS is a widely used approach in investigating PPIs. Although the co-IP method reveals the proteins that physically and stably bind to the protein of interest, the resulting MS data are frequently accompanied with nonspecific binders arising from mild washing conditions. Moreover, transient and/or weak PPIs are often missed since their bindings could not survive the cell lysis or washing steps. To overcome these limitations, scientists have developed complementary approaches based on proximity labeling for better interactome profiling.

Among the emerging proximity labeling methods, the APEX-based approach pioneered by Ting et al. (2013) and Han et al. (2019) is one of the most employed. The engineered APEX enzyme is genetically encoded to express in frame with the protein of interest. Upon transient H_2_O_2_ exposure, the enzyme immediately (<1 min) converts biotin phenol into a phenoxyl radical, which attacks nearby electron-rich amino acids such as tyrosine. In this regard, the proteins within a radius of 10–20 nm to the bait protein are covalently labeled with biotin tags. Those labeled proteins can be purified using streptavidin-conjugated beads. Given the strong biotin-streptavidin interaction, nonspecific binding proteins can be efficiently removed by using harsh washing conditions (e.g., with a urea-containing buffer).

Aside from APEX, biotin protein ligase (BirA) from *Escherichia coli* could be fused with the protein of interest to convert biotin and ATP to biotinyl-AMP ester that reacts with lysine residues in nearby proteins. The engineered BirA was pioneered by Choi-Rhee, Schulman, and Cronan (2004) in the early 2000s through introducing an R118G mutation, which provides enhanced affinity with biotinyl-AMP, thereby offering improved selectivity for labeling only proteins in close proximity. In 2011, Roux et al. (2012) streamlined the BirA-R118G-based approach for efficient proximity labeling in living cells, termed BioID, coupled with MS to systematically investigate protein interaction networks. To date, BioID has been applied to identify the interactomes of many important cellular proteins.

Proximity labeling has also been successfully applied to RNA-binding proteins. Han et al. (2020) fused the engineered APEX to Cas13 (Cas13), an engineered enzyme that recognizes specific RNA through user-designed guide RNA. Consequently, the Cas13-fused APEX labeled the proteins near the RNA motif of interest, and those RNA-binding proteins could be identified and quantified through proteomic analysis. In their proof-of-concept investigation, the peroxidase was subsequently targeted to the telomerase RNAs to identify novel binders such as m^6^A demethylase ALKBH5, which removes the m^6^A modification on telomerase RNA and subsequently regulate telomerase assembly and downstream functions. Alternatively, RNA of interest could be genetically engineered to fuse with a sequence encoding the bacteriophage MS2. Through fusing APEX or biotin ligase with MS2 coat protein (MCP) that binds specifically to MS2, the labeling enzyme could be guided to the RNA for labeling RNA-binding proteins (Tutucci et al. 2018).

To effectively employ proximity labeling for investigating the cellular regulation of epitranscriptomic RWE proteins, careful selection of the appropriate enzyme is critical. For fast, high-resolution snapshots of epitranscriptomic regulations, the APEX-based approach is ideal due to its rapid labeling abilities, capturing transient interactions within seconds. Conversely, BioID is better suited for studying sustained or slower interactions, as they allow labeling over a span of hours to days. Depending on the biological question under investigation, proper labeling strategy and experimental design should be employed to examine epitranscriptomic RWE proteins.

The labeling enzyme should be fused with the RWE protein of interest in a position that preserves its function and localization, ensuring that its native activity and interactions remain intact. For example, METTL3 forms a heterodimer with METTL14 via its C-terminal domain, making it advisable to avoid fusing the labeling enzyme at its C-terminus, as this could disrupt complex formation (Liu et al. 2014; Wang, Doxtader, and Nam 2016). Depending on the structural and functional characteristics of the protein of interest, it may be necessary to test both N- and C-terminal fusions to determine the optimal configuration that maintains the target protein’s cellular functions.

The linker between the RWE protein and the labeling enzyme is another critical factor (Chen, Zaro, and Shen 2013). A longer linker can reduce steric hindrance, better preserve the protein’s native function, and provide a larger labeling radius, which may be useful for capturing more distal interacting partners. However, longer linkers may also increase nonspecific labeling, reducing precision (Qin et al. 2021). On the other hand, a shorter linker offers more precise labeling but may interfere with the proper folding or complex formation of the RWE proteins, potentially skewing the results. When designing proximity labeling experiments in epitranscriptomic studies, it’s important to strike a balance between preserving the native behavior of RWE proteins and achieving sufficient labeling specificity. Proper experimental design and pilot tests to evaluate fusion strategies and linker lengths will optimize labeling efficiency while minimizing artifacts.

Optimizing expression levels for proximity labeling experiments involving epitranscriptomic RWE proteins follows principles similar to the aforementioned co-IP experiments. Expressing the fusion protein at endogenous levels is ideal for capturing physiologically relevant interactions. However, endogenous expression may result in weak or undetectable labeling, especially if the proteins of interest a expressed at low levels. Overexpression of the fusion protein, on the other hand, can lead to artificial interactions and potentially distort the native regulation of RWE proteins.

To avoid these artifacts, proximity labeling is best performed with genetic tagging of the RWE protein at its endogenous locus, ensuring that labeling is reflective of native protein interactions. If the native expression level of the RWE protein is too low for sufficient labeling, controlled overexpression may be necessary. In such cases, it’s crucial to optimize the expression levels to ensure minimal perturbation of cellular processes. The goal is to achieve the lowest possible expression level of the fusion protein while allowing for efficient labeling detectable by MS.

While proximity labeling provides a highly specific means to identify interacting partners of RWE proteins, nonspecific labeling of abundant proteins that are not part of the actual protein complexes can still occur, potentially leading to false positives. To address this, proper negative controls are essential to differentiate background labeling from those arising from specific interactions. A common approach is to express the labeling enzyme alone or its fusion with a protein not present endogenously (e.g., GFP), and targeted to the same subcellular compartment (e.g., nucleus, RNA granules) (Han et al. 2020). In addition, the expression of the control group labeling enzyme should be at a similar level of the fusion RWE protein to correct for nonspecific labeling. This enables the subtraction of background signals caused by highly abundant or nonspecific proteins. Detailed guidance on designing effective negative controls is discussed previously (Cho, Branon, Udeshi, et al. 2020).

#### 4.2.3 | Transient and Stable Expression

The fusion proteins for co-IP or proximity labeling experiments can be transiently or stably overexpressed in cells, with each method offering different advantages and challenges. Transient expression, typically achieved through plasmid transfection, introduces the gene encoding the fusion protein with a user-selected promoter to tune the expression level for a short period, usually lasting 1–3 days (Ishii, Okahata, and Sato 2001; Stepanenko and Heng 2017). This approach is efficient in generating high levels of the fusion protein, which can enhance labeling efficiency and render it easier to detect interactions. In addition, transient expression systems are often easy to establish and modify, offering flexibility in experimental design and allowing for rapid testing of various conditions.

Transient expression, however, often results in protein levels far exceeding that of the endogenous counterparts, which can perturb native PPIs, leading to artifacts that do not reflect normal physiological conditions. Additionally, it can be difficult to maintain consistent expression levels across experimental replicates, introducing variability that compromises the precision of the data. Given that epitranscriptomic RWE proteins regulate protein expression through RNA modifications, overexpression of these RWE proteins can significantly disrupt the cellular proteome. For example, overexpressing METTL3 or YTHDF1 may result in elevated m^6^A levels in cellular RNA or impaired binding of other proteins to m^6^A-containing RNA, thereby altering normal mRNA processing, translation, and protein abundance. Such reprogramming of the proteome may yield misleading results and obscure the true regulatory mechanisms of RWE proteins.

Stable expression, on the other hand, involves integrating the gene encoding the fusion protein into the genome, allowing for continuous and long-term protein expression. While generating a stable expression system—typically with the use of lentiviral vectors—can be more time-consuming and labor-intensive, it offers tighter control over protein levels and greater reproducibility across experimental replicates (Cockrell and Kafri 2007; Dull et al. 1998). Stable expression systems can often be fine-tuned to express proteins at near-physiological levels, reducing the risk of artifacts accompanied with transient overexpression. However, over time, cells may adapt to RWE protein overexpression by altering their proteomic or regulatory landscapes, which can also lead to nonphysiological interactions or changes in cellular functions. This adaptive response can obscure the true nature of the PPIs under study.

To mitigate these risks, careful experimental design and proper controls are essential. It is crucial to balance the need for detectable PPIs with the risk of altering normal cellular processes. Strategies such as optimizing expression levels to minimize overexpression-induced artifacts, using inducible systems, or employing CRISPR-mediated knock-in approaches to tag the protein of interest at endogenous levels can help preserve the biological relevance of the findings (Banan 2020; Kong and Chan 2023). Cross-validation using complementary techniques, for example, endogenous protein interaction studies, can also strengthen the validity of the results.

## 5 | Identification of Novel Epitranscriptomic Reader Proteins

### 5.1 | Affinity-Based Approaches to Discover Epitranscriptomic Reader Proteins

Reader proteins bind to chemically modified RNA, playing a pivotal role in executing downstream regulatory processes (Du, Zhao, et al. 2016; Shi et al. 2017). Identifying these proteins is essential for understanding the mechanisms of epitranscriptomic regulation. To discover such binding proteins, an affinity enrichment approach is often utilized. This method involves using RNA probes carrying specific chemical modifications on the sequence of interest as baits to enrich binding proteins, where the RNA probes also contain an affinity handle to enable enrichment. The proteins bound to these probes are then processed for discovery or targeted proteomic analysis.

**FIGURE 4 |:**
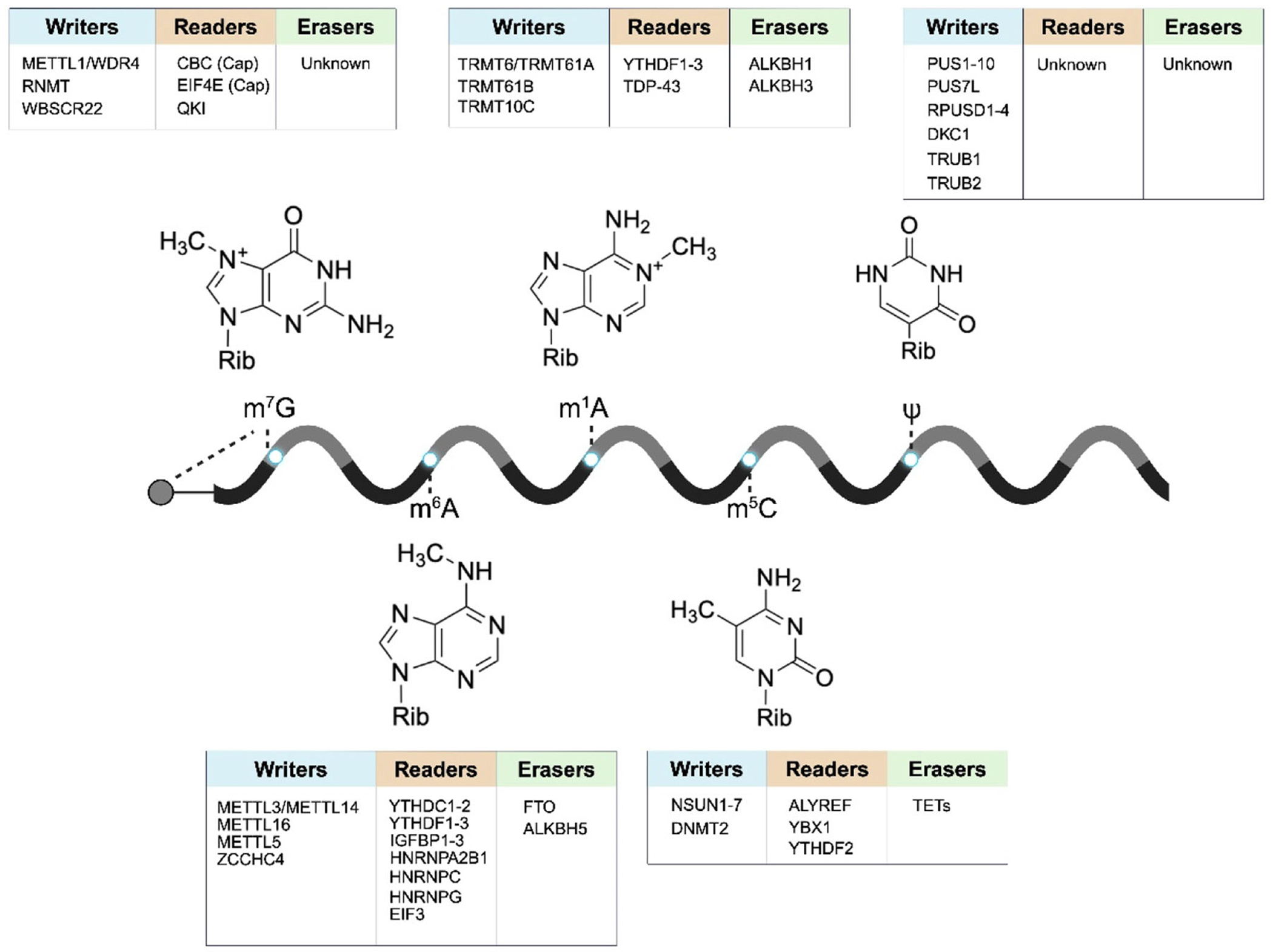
A summary of the major reader, writer, and eraser proteins of common modified nucleosides in RNA, including m^6^A, m^1^A, m^5^C, m^7^G, and Ψ.

To investigate the role of m^1^A in RNA, Dai et al. (2018) employed a 5’-biotin-labeled RNA sequence derived from the human *SOX18* gene, which carried the m^1^A modification, as a bait to enrich m^1^A-binding proteins. Notably, in affinity enrichment studies, it is crucial to include a control to validate the specificity of the observed interactions. For the investigation into m^1^A-binding proteins, a bait of the same RNA sequence but without m^1^A served as the negative control, which allowed for discerning specific interactions attributable to the m^1^A modification. This complex was subsequently analyzed using SILAC-based quantitative proteomics. The results from such analysis revealed that YTHDF1–3 and YTHDC1 proteins bind to m^1^A in RNA, whereas YTHDC2 does not exhibit apparent binding affinity toward m^1^A-containing RNA (Figure 4). Moreover, TDP-43 was found to be an m^1^A reader and this interaction was shown to trigger neurodegeneration in CAG repeat expansion diseases (Sun et al. 2023). A similar approach led to the identification of YTHDF2 as 5-methylcytidine (m^5^C)-binding protein, and this binding modulates rRNA maturation (Dai et al. 2020). Moreover, a recent study also led to the discovery QKI family proteins as readers for m^7^G in mRNA (Zhao et al. 2023).

### 5.2 | Chemoproteomics Approach to Discover Epitranscriptomic Reader Proteins

Chemoproteomics, which combines chemical biology with MS-based proteomics, has emerged as a powerful tool to study epitranscriptomic reader proteins. In this approach, modified nucleoside-containing RNA probes form covalent bonds with target proteins through techniques such as activity-based protein profiling (ABPP) or photo-crosslinking. The covalently tagged proteins can then be pulled down for MS analysis. The covalent nature of the interaction between the RNA probe and reader proteins allows for more stringent washing conditions during enrichment, thereby significantly improving the specificity of the captured interactions. Moreover, the rapid reaction kinetics provided by the chemoproteomic approach makes it possible to identify transient and/or weak protein–RNA interactions, which are often missed when using noncovalent binding approaches. Thus, chemoproteomics is valuable for capturing dynamic and short-lived RNA–protein interactions.

Heindel et al. (2023) harnessed photo-crosslinking to enable the formation of covalent bonds between RNA baits and their binding proteins. The formation of covalent bonds allows for the use of more rigorous washing conditions to remove nonspecifically bound proteins, thereby enhancing assay selectivity. In this context, it is of note that nonspecific species of high abundance can diminish ionization efficiency and negatively impact the dynamic range of analysis. Additionally, the MS^2^ of peptides originated from true binders, owing to their relatively low abundances, might not be collected in DDA experiments. Hence, efficient removal of nonspecific binders in the photo-crosslinking approach in principle allows for improved sensitivity, selectivity, and quantitation accuracy in proteomic identification of epitranscriptomic readers.

Huang et al. (2021) employed a DNA-guided photoactivation-based chemoproteomics technique to enrich m^6^A-binding proteins, which were subsequently digested on-beads and analyzed using label-free DIA analysis. This approach facilitated the isolation of a wide array of known and new m^6^A-associated RNA-binding proteins. The quantitative proteomics data revealed established m^6^A-binding proteins, for example, YTHDF1-3, YTHDC2, and RBM39, and a new reader, DDX1. The latter is an ATP-dependent RNA helicase. Hence, this result suggests a potential m^6^A-dependent mechanism in helicase-mediated RNA unwinding, indicating a novel functional role of m^6^A in RNA metabolism. Notably, while chemoproteomics has been largely employed to identify reader proteins, it can also be utilized for the identification of writer and eraser proteins (Dai, Yu, and Kleiner 2023). Together, chemoproteomics constitutes a high-throughput, highly accurate, and sensitive tool for unbiased identification of epitranscriptomic reader proteins.

## 6 | Quantitative Assessment of Epitranscriptomic RWE Proteins

The expression regulation of RWE proteins is of keen interest within the epitranscriptomic community, as aberrant expression of these proteins has been linked with numerous diseases, including cancer and neurodegenerative disorders. However, the substantial differences in the dynamic range of RWE proteins across samples pose significant challenges for their reproducible identification and quantification. Addressing these challenges remains a critical focus in the epitranscriptomics field.

To overcome challenges in sensitivity and reproducibility, targeted proteomics approaches have been developed for assessing the differential expression of epitranscriptomic RWE proteins. Qi, Miao, and Wang (2022) and Qi et al. (2023) developed a scheduled PRM-based proteomics strategy that integrates with SILAC-based quantitation. This approach enabled the successful profiling of a total of 152 epitranscriptomic RWE proteins in a single injection, demonstrating the high throughput and sensitivity of the method. Furthermore, the LC-PRM analysis revealed significant insights, such as the association of TRMT1, an m^2,2^G writer protein, with radioresistance in breast cancer (Qi, Miao, and Wang 2022).

While SILAC offers excellent accuracy for relative quantitation, its application is limited in animal studies or clinical samples, as discussed above. To address this limitation, Qi, Liu, et al. (2022) developed a scheduled LC-PRM platform that utilizes SIL peptides as internal or surrogate standards for precise profiling of epitranscriptomic RWE proteins. Employing SIL-based LC-PRM analysis, Yin et al. (2023) successfully profiled the expression of epitranscriptomic RWE proteins over the course of osteogenic differentiation of human embryonic stem cells—a situation where SILAC labeling proves difficult. This sensitive LC-PRM analysis allowed for the identification of 127 RWE proteins, and the work underscored the role of METTL1 in regulating osteogenic differentiation through the cytokine network, demonstrating the platform’s utility and versatility in complex biological investigations (Yin et al. 2023).

Together, targeted proteomics analysis serves as a powerful tool for the systematic interrogation of the expression of epitranscriptomic RWE proteins, offering unprecedented sensitivity, reproducibility, and throughput. Furthermore, integration of stable isotope labeling into the method enables accurate quantitative assessments across various sample types. This combination enhances the analytical precision and broadens the scope of their applications in epitranscriptomic studies.

## 7 | PTMs of Epitranscriptomic RWE Proteins

Proteins can undergo PTMs to meet spatial and temporal demands within the cell. These modifications, including acetylation, methylation, phosphorylation, and so forth, provide an additional layer of regulation, allowing for the functional and spatial modulation of proteins without the need for changes at the transcriptional and translational levels. These PTMs are typically reversible, which facilitates rapid alterations in protein activity in response to various stimuli. Notably, PTMs often exhibit low stoichiometry, and identical modifications may occur at multiple sites within the same protein. Given these complexities, MS has emerged as the premium technique for conducting high-throughput and precise analyses of PTMs.

Phosphorylation, a prevalent form of PTM in cellular processes, is catalyzed by kinases. Previously, Sun and Li (2013) discovered, from MS analysis, that METTL3 undergoes phosphorylation at Ser-43, Ser-50, and Ser-525 by the ERK pathway. Additionally, the ERK pathway has been shown to phosphorylate WTAP at Ser-306 and Ser-341. These phosphorylations promote the deubiquitylation of METTL3 and WTAP, thereby stabilizing the m^6^A methyltransferase complex.

The study of PTMs via MS often necessitates the enrichment of samples due to the low modification stoichiometry. Additionally, chromatography must be meticulously optimized to align with the physical properties of the PTMs under investigation. Achieving sufficient chromatographic resolution and high-quality spectral data is essential for unambiguous PTM identification. This optimization ensures the accuracy and reliability of MS analysis in detecting and characterizing these critical molecular alterations. The discovery of PTMs of epitranscriptomic regulatory proteins using MS is discussed in detail elsewhere (Zhang, Wang, and Li 2010).

## 8 | Functional Characterizations of Epitranscriptomic RWE Proteins

Epitranscriptomic RWE proteins play crucial roles in modulating RNA modifications that subsequently influence gene expression within the cell. These proteins regulate chemical modifications in RNA, which can in turn modulate gene expression. Direct assessment of changes in protein expression modulated by the epitranscriptomic RWE proteins provides critical information about the functions of these epitranscriptomic modulators. To understand the functions of these RWE proteins, genetic manipulations of specific proteins followed by global proteomics analysis has emerged as a powerful tool due to its high throughput and accuracy (Rabia et al. 2018).

Peng et al. (2022) conducted an investigation where METTL3 was overexpressed in gastric cancer cells, followed by an examination of the consequent changes in protein expression using TMT-based high-throughput quantitative proteomics analysis. They found that METTL3 assumes a regulatory role in mitochondrial metabolism in gastric cancer cells. Similarly, one can introduce mutations into epitranscriptomic regulatory proteins within cells and investigate the functional consequences of these mutations using proteomics. For instance, Zhang et al. (2023) introduced a gain-of-function missense mutation, R298P, into METTL14 and observed, through proteomics analysis, that the mutation results in diminished expression of proteins involved in WNT signaling.

While global proteomic analysis is invaluable for providing a comprehensive snapshot of differential protein expression across the entire proteome, its sensitivity in detecting low-abundance proteins can be limited. To address this challenge, targeted proteomics approaches, including MRM and PRM, are employed to enhance the sensitivity and depth of coverage for specific subsets of proteome of interest. Our laboratory utilized a high-throughput scheduled LC-MRM method, in conjunction with synthetic SIL peptides, to assess systematically the changes in the small GTPase proteome that arise from genetic depletions of m^6^A RWE proteins (Yang et al. 2020). These alterations stem from the depletion of m^6^A methyltransferase METTL3, demethylases (ALKBH5 and FTO), and m^6^A reader proteins (YTHDF1, YTHDF2, and YTHDF3). The use of a triple-quadrupole mass spectrometer in the MRM mode enabled us to consistently quantify over 50% of the small GTPase proteome across the various knockout cell lines. The results from this study demonstrate the exceptional sensitivity and reproducibility of the targeted proteomic approach in revealing the critical impact of m^6^A on posttranscriptional regulation of the small GTPase proteome.

These examples collectively demonstrate that MS-based proteomic analysis, when integrated with genetic manipulation of epitranscriptomic RWE proteins, constitutes a highly effective tool for systematically identifying the target proteins regulated by epitranscriptomic modification. It can be envisaged that a similar approach can be employed to examine other subsets of the proteome that are regulated by m^6^A and other modified nucleosides in RNA.

## 9 | Conclusions and Outlook

**FIGURE 5 |:**
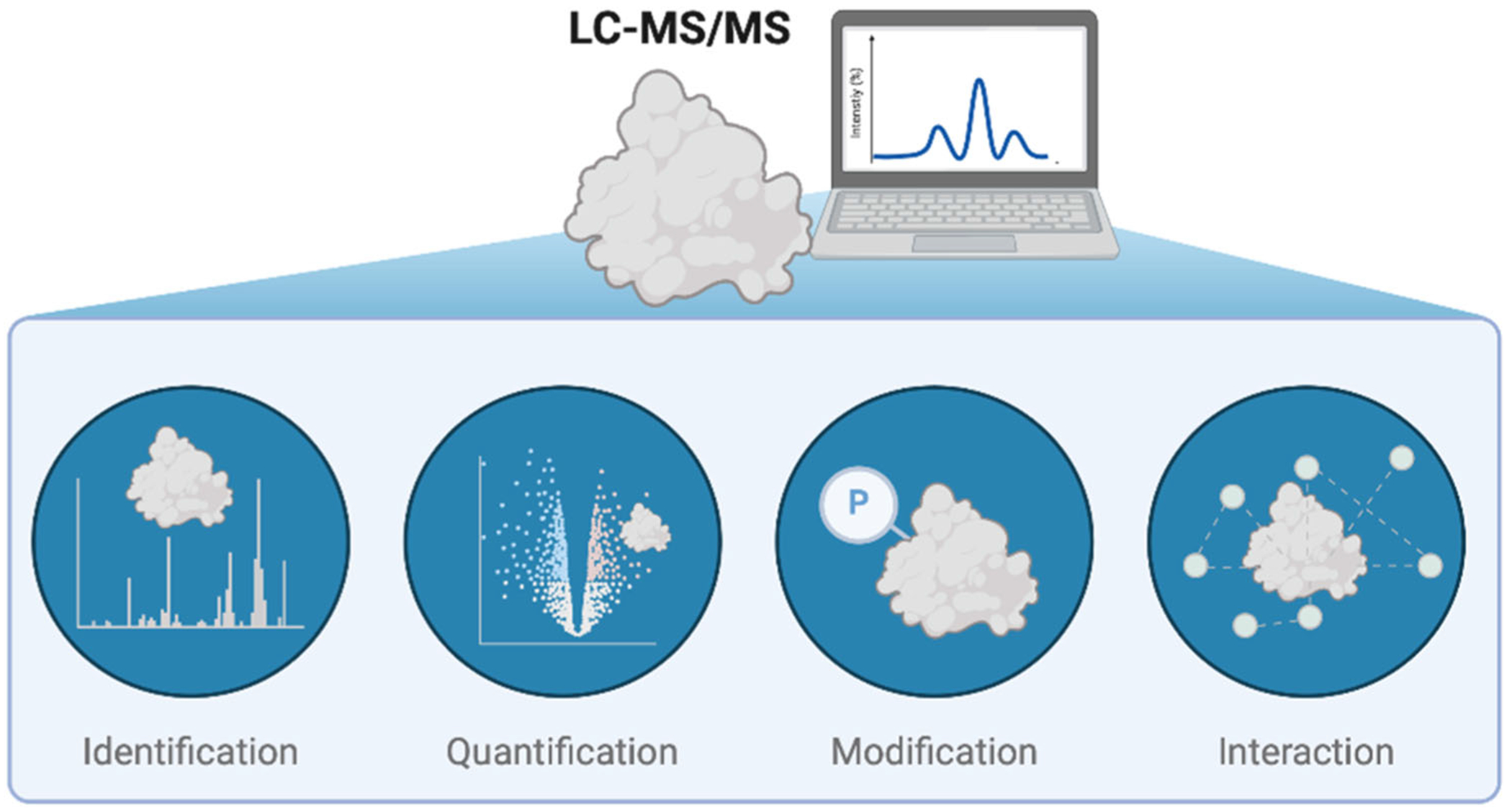
MS provides precise identification and quantification of epitranscriptomic regulatory proteins and their PTMs.

Epitranscriptomic regulation plays a pivotal role in enabling rapid and reversible changes at both the transcriptional and translational levels, thereby modulating cellular physiology. Recently, novel RNA modifications, for example, glycation, and their associated reader proteins have been continuously discovered, suggesting that our current understanding of epitranscriptomics represents only the tip of the iceberg with many more regulatory mechanisms yet to be uncovered (Flynn et al. 2021; Perr et al. 2023; Xie et al. 2024; Zhang et al. 2024). MS-based proteomics has been employed in many aspects of epitranscriptomics research, including the identification of novel RWE proteins, the quantifications of these proteins, as well as the characterizations of their PTMs, interaction partners, and biological functions (Figure 5). With continuous improvements in instrumentation and sample preparation methods, MS is poised to sustain its critical role in delivering high-throughput and precise data about epitranscriptomic RWE proteins and their interactions and regulations, which will further advance our understanding of epitranscriptomics.

## Abbreviations:

ALKBH5: alkB homologue 5
DDA: data-dependent acquisition
DIA: data-independent acquisition
eIF3: eukaryotic initiation factor 3
FTO: fat mass and obesity-associated protein
hnRNPC: heterogeneous nuclear ribonucleoprotein C
ICAT: isotope-coded affinity tag
iTRAQ: isobaric tags for relative and absolute quantitation
METTL3: methyltransferase-like 3
METTL14: methyltransferase-like 14
METTL16: methyltransferase-like 16
mRNA: messenger RNA
